# Cost-effectiveness analysis of repeated self-sampling for HPV testing in primary cervical screening: a randomized study

**DOI:** 10.1186/s12885-020-07085-9

**Published:** 2020-07-13

**Authors:** Riina Aarnio, Ellinor Östensson, Matts Olovsson, Inger Gustavsson, Ulf Gyllensten

**Affiliations:** 1 Department of Women’s and Children’s Health, Uppsala University, 751 85 Uppsala, Sweden; 2 Department of Women’s and Children’s Health, Karolinska Institutet, Tomtebodavägen 18A, 171 77 Stockholm, Sweden; 3 Department of Medical Epidemiology and Biostatistics, Karolinska Institutet, Nobels väg 12A, 171 65 Stockholm, Sweden; 4 Department of Immunology, Genetics, and Pathology, Biomedical Center, SciLifeLab Uppsala, Uppsala University, Box 815, 75108 Uppsala, Sweden

**Keywords:** Self-sampling, HPV testing, Primary cervical screening, Cost-effectiveness, CIN2 +, Precancerous lesion, Cervical cancer

## Abstract

**Background:**

Human papillomavirus (HPV) testing is recommended in primary cervical screening to improve cancer prevention. An advantage of HPV testing is that it can be performed on self-samples, which could increase population coverage and result in a more efficient strategy to identify women at risk of developing cervical cancer. Our objective was to assess whether repeated self-sampling for HPV testing is cost-effective in comparison with Pap smear cytology for detection of cervical intraepithelial neoplasia grade 2 or more (CIN2+) in increasing participation rate in primary cervical screening.

**Methods:**

A cost-effectiveness analysis (CEA) was performed on data from a previously published randomized clinical study including 36,390 women aged 30–49 years. Participants were randomized either to perform repeated self-sampling of vaginal fluid for HPV testing (*n* = 17,997, HPV self-sampling arm) or to midwife-collected Pap smears for cytological analysis (*n* = 18,393, Pap smear arm).

**Results:**

Self-sampling for HPV testing led to 1633 more screened women and 107 more histologically diagnosed CIN2+ at a lower cost vs. midwife-collected Pap smears (€ 229,446 vs. € 782,772).

**Conclusions:**

This study resulted in that repeated self-sampling for HPV testing increased participation and detection of CIN2+ at a lower cost than midwife-collected Pap smears in primary cervical screening. Offering women a home-based self-sampling may therefore be a more cost-effective alternative than clinic-based screening.

**Trial registration:**

Not registered since this trial is a secondary analysis of an earlier published study (Gustavsson et al., British journal of cancer. 118:896-904, 2018).

## Background

Organized screening with Papanicolaou cytology (Pap smear) has resulted in a major reduction in both the incidence of cervical cancer and related mortality [1]. Nevertheless, about 500 women are diagnosed with cervical cancer, and about 140 women die of it every year in Sweden [2]. *Persistent* infection with oncogenic high-risk types of human papillomavirus (HPV) is a prerequisite for the development of cervical cancer [3], although most HPV infections clear spontaneously, with no increased risk for cervical cancer. HPV testing has greater sensitivity in revealing histological cervical intraepithelial neoplasia grade 2 or more (CIN2+) than cytology [4, 5], and primary cervical screening by means of HPV testing is recommended in Europe [6, 7]. Because of the lower specificity of HPV testing, cytological testing of HPV-positive samples at primary screening (cytology triage) is recommended. This presents a challenge in how to manage women that are HPV-positive but cytology-negative, since they have an elevated risk of CIN2+ [8]. In a previous study, we proposed that repeated HPV testing to identify persistent infections can be used as an alternative to cytology triage [9]. Among women with an HPV-positive screening result, 70% had an HPV infection when retested 4–6 months later, and repeated HPV testing is estimated to result in similar overall specificity as with cytology based screening.

A low population coverage of screening has been identified as an important risk factor of incident cervical cancer [10]. Previous studies have revealed increased response rates among non-responders by offering self-sampling kits for HPV testing, vs. other options [11–16]. In a large meta-analysis self-sampling for primary cervical screening was recommended, but only when using PCR-based HPV tests [17].

An important criterion for a screening program is the cost-effectiveness (https://www.socialstyrelsen.se/globalassets/sharepoint-dokument/artikelkatalog/nationella-screeningprogram/2019-4-12.pdf), and health-economic evaluations are therefore needed before deciding on implementation of new screening tests or strategies. The first aim of this study was to compare the cost-effectiveness of repeated self-sampling for HPV testing with midwife-collected Pap smear cytology based on data from a recent randomized study on primary cervical screening [18]. The second aim was to estimate the cost of treatment and follow-up of histological CIN2 + .

## Methods

### Study design

**Fig. 1:**
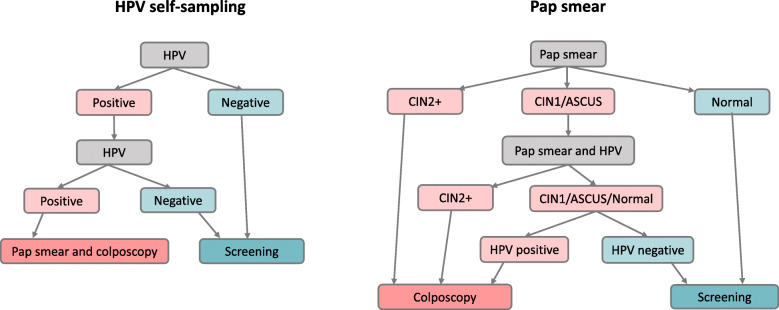
Study protocol flowchart in the HPV self-sampling and Pap smear arms

**Fig. 2:**
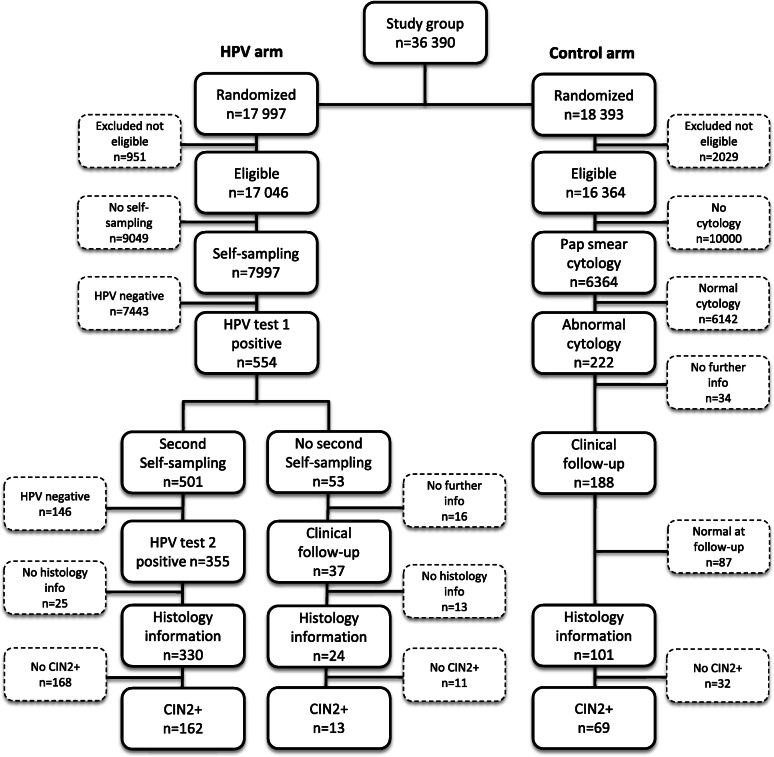
Study flowchart with number of women at different steps in the HPV self-sampling and Pap smear arms

This study is a secondary analysis based on clinical and cost data from a previously published randomized study [18]. During 2013–2015 a total of 36,390 women aged 30–49 years planned for regular screening invitation in Uppsala, Sweden, were randomized into two arms; a) repeated self-sampling of vaginal fluid for HPV testing (*n* = 17,997, HPV self-sampling arm) or to midwife-collected Pap smear for cytological analysis (*n* = 18,393, Pap smear arm) [18]. The study flowchart is shown in Fig. 1 and number of women included are shown in Fig. 2. Women with previous hysterectomy or current pregnancy were recommended in the invitation letter not to participate in the study. Some of these women nevertheless performed self-sampling and women with previous hysterectomy were excluded after second self-sampling, while the pregnant women were included in this study. A cost-effective analysis (CEA) was performed comparing the alternative screening strategies, based on quantification, effectiveness and cost data. Additional treatment and follow-up data were collected from patient files.

### HPV self-sampling arm

Women were sent an invitation with a self-sampling kit for vaginal fluid, including a sampling brush, an FTA card, a step-by-step guide on how to perform the sampling and a pre-addressed and postage-paid return envelope. Women performed the self-sampling and sent the FTA card to the Department of Immunology, Genetics and Pathology at Uppsala University (HPV laboratory) for HPV testing. Women with a positive HPV test result were sent a second self-sampling kit for repeat sampling after 3–6 months. Participation in primary screening was classified as complete when having an analyzed first negative HPV test, or in the case of a first positive HPV test, after repeated HPV testing. Women with two consecutive HPV-positive self-samples were referred to colposcopy. All HPV-negative women were referred back to screening (Fig. 1). Details on sampling material, processing and HPV analysis are described in the published study [18].

### Pap smear arm

Women were sent an invitation to schedule an appointment at the local midwife clinic, where Pap smear sampling for cytology was performed. Participation in primary screening was classified complete when having an analyzed Pap smear. Women with CIN2+ in cytology were referred to colposcopy. Women with low-grade cervical intraepithelial neoplasia (CIN1) or atypical squamous cells of unknown significance (ASCUS) in cytology were scheduled for repeated Pap smear and HPV test by a midwife after 3 months. HPV-positive women and women with CIN2+ in cytology were referred to colposcopy, while HPV-negative women without CIN2+ in cytology were referred back to screening (Fig. 1). Cytology and histology were performed at the Department of Pathology and Cytology, Uppsala University Hospital. Pap smears and histological diagnoses were classified according to CIN terminology. The highest histological grade found in each patient was used for interpretation.

### Colposcopy

Women with repeated HPV-positive results, ASCUS/CIN1 cytology and HPV-positive result or CIN2+ cytology were followed up by colposcopy. Here, the squamocolumnar junction and transformation zone were identified, 5% acetic acid and iodine solution were applicated and after visual evaluation, all lesions were biopsied. A Pap smear was collected on all HPV-positive women in the self-sample arm. In cases of transformation zone 3 (TZ3) a sample was also collected for endocervical cytology. Mainly one expert colposcopist performed colposcopies among women in the HPV self-sampling arm, while different colposcopists performed colposcopies in the Pap smear arm.

### Treatment of precancerous lesions and cancers

Women with histological CIN2+ were treated according to current clinical recommendations. In the Pap smear arm, about one fifth of the women with CIN were treated at a regional hospital (Enköping lasarett, Enköping). The rest of the women with CIN and women with cancer were treated at the Department of Gynecology and Obstetrics, Uppsala University Hospital. Precancerous lesions and micro-invasive cancers were treated by using the loop electrosurgical excision procedure (LEEP), most of them under local anesthesia but some under general anesthesia. Treated women were invited for a follow-up appointment (‘test of cure’) with a midwife or a gynecologist in 4–6 months. At this appointment, the midwives collected a Pap smear and a sample for HPV testing, and in addition to them, the gynecologist also carried out colposcopy. The cancer cases were discussed at a multidisciplinary meeting after requisite radiological investigation, usually chest and abdominal CT scans and a pelvic MR scan. Surgical treatment consisted of either simple or radical hysterectomy or trachelectomy. Radical surgery included excision of the upper vagina and parametria with bilateral pelvic lymphadenectomy beyond removal of the uterus (hysterectomy) or the cervix (trachelectomy). Surgery was performed either by laparotomy or in most cases by means of minimally invasive techniques, such as laparoscopy or robotic-assisted laparoscopic surgery.

### Outcome data

Clinical data (at the time of screening invitation and the cytological and histological test results at clinical follow-up) were retrieved from a database at the Department of Pathology and Cytology, Uppsala University Hospital. All events from invitation until diagnosis were noted for each patient in both study arms. The treatment records, including further preoperative assessment and follow-up after treatment in cases of CIN2+, were manually checked in the patient files until 31 December 2018. All events were included after LEEP until the ‘test of cure’ was accepted (HPV-negative and Pap smear cytology <CIN2), or after surgical treatment of cancer, until the first postoperative visit. Possible treatments and follow-up in cases of CIN1 were not included in this analysis.

### Cost-effectiveness analysis (CEA) and cost estimation

A CEA was performed using healthcare provider perspective [19]. The unit costs for each screening event were retrieved from the HPV laboratory and Uppsala region financial records. Direct medical costs of inpatient and outpatient healthcare were retrieved from the financial records at Uppsala University Hospital. When needed costs were adjusted for inflation by using the consumer *price* index (CPI) [20] and converted to 2019 Euros (mean annual exchange rate, € 1 = 10.5912 SEK). A cost per screened woman was calculated in each study arm according to the study protocol. Screening strategies (HPV self-sampling vs. Pap smear) were ranked from the lowest to the most costly. Incremental cost-effectiveness ratios (ICERs) per extra screened women were calculated by dividing the cost difference (cost) with the difference in number of screened women (effect) between the two screening arms in the randomized study. At clinical follow-up, also the ICERs per extra detected woman with CIN2+ were calculated. If a screening arm was more costly and less effective than the comparative one, it was defined as strongly dominated. A sensitivity analysis was performed to account for the uncertainty of screen participation and trends in direct medical costs. Moreover, using the cost data we estimated the cost of treatment and follow-up of histological CIN2 + .

## Results

### Cost-effectiveness analysis on primary screening including clinical follow-up

The participation rate in cervical screening was significantly higher in the HPV self-sampling arm than in the Pap smear arm (47% vs. 39%, *P* < 0.001). In the HPV self-sampling arm, 7997 women returned a self-sample for HPV testing, of which 7443 (93%) were HPV-negative and considered as completely screened (Table 1). A second self-sampling kit was sent to 554 HPV-positive women and 501 (90.4%) women returned a sample and considered completely screened. In the second HPV test, 355 women were positive and were referred to colposcopy. In total, 175 cases of histological CIN2+ were identified in the HPV self-sampling arm. In the Pap smear arm, 6364 women visited a midwife for a Pap smear and considered completely screened. Among these 6142 (97%) had normal cytology, whereas 222 women had abnormal Pap smear results and where referred to follow-up. In total, 68 women with histological CIN2+ were identified in the Pap smear arm (Table 1). All ten cancer cases in the HPV self-sampling arm had FIGO stages 1A1-1B1 without cancer recurrence in May 2020. Of the five cancer cases in the Pap smear arm four had FIGO stages 1A1-1B1 without cancer recurrence in May 2020, while one woman with FIGO stage 1B2 had died because of her cervical cancer. Thus, there seems to be no increase in risk for more advanced cancer due to the time span between first and second HPV test.

**Table 1:** Resources required per screened woman by intervention arm, with associated costs in 2019 (€ 1 = 10.5912 SEK)

		**Pap smear (*n*** **= 18,393)**	**HPV self-sampling (*n*** **= 17,997)**		
	**Unit Cost (€)**	**Units**	**Costs (€)**	**Units**	**Costs (€)**
**Primary screening**					
Pap smear cytological analysis	25	6364	159,100		
Midwife appointment for sampling	98	6364	623,672		
First HPV self-sampling kit, return and HPV test (including postal fees)	27			7997	215,919
2nd HPV self-sampling kit, return and HPV test (including postal fees)	27			501	13,527
**Total cost of primary screening**			**782,772**		**229,446**
Screened women		6364		7997	
Abnormal cytology in need of clinical follow-up		222			
2nd HPV-positive in need of clinical follow-up				355	
Incremental effect (screened women)			1633		
Cost per screened woman			123		29
ICER per extra screened woman			− 339		
**Clinical follow-up**					
Pap smear cytological analysis	25	238	5950	428	10,700
HPV analysis*	38	173	6574	46	1748
Midwife appointment for sampling	98	166	16,268	4	392
Colposcopy appointment	470	114	53,580	384	180,480
Biopsy histological analysis	147	114	16,758	367	53,949
**Total cost of clinical follow-up**			**99,130**		**247,269**
**Total cost of primary screening + clinical follow-up**			**881,902**		**476,715**
CIN2+		68		175	
Incremental effect (CIN2+)			107		
Cost per woman with CIN2+			12,969		2724
ICER per extra detected woman with CIN2+			− 3787		
**Sensitivity analysis HPV vs. Pap smear (ICER per extra detected woman with CIN2+)**					
***Efficacy parameters***					
Participation rate + 25% Pap smear		*7955*	−5451		
Participation rate + 25% HPV self-sampling			− 3124	*9996*	
Participation rate − 25% Pap smear		*4773*	−111		
Participation rate − 25% HPV self-sampling			− 4129	*5998*	
***Screening cost variation***					
HPV self-sampling + 25%	*34*		− 3092		
HPV self-sampling −25%	*20*		− 4160		
Pap smear cytological analysis + 25%	*32*		− 3990		
Pap smear cytological analysis −25%	*19*		− 3262		
Midwife appointment + 25%	*122*		− 5076		
Midwife appointment −25%	*73*		− 2176		
Midwife appointment −50%	*49*		− 727		

*Total cost of HPV test including HPV kit and analysis performed at the HPV laboratory, Uppsala University and transfer of the results to the database at the Department of Cytology and Pathology, Uppsala University Hospital

The total cost of primary screening was higher for the Pap smear arm than for the HPV self-sampling arm (€ 782,772 vs. € 229,446), and the Pap smear arm was thus strongly dominated (Table 1). The HPV self-sampling arm also resulted in detection of more cases of CIN2+ at a lower cost in comparison with Pap smear arm and is a cost-saving alternative (clinical follow-up, Table 1). Sensitivity analysis for participation rate, screening test cost (Pap smear analysis and HPV test analysis), self-sampling kit cost and midwife appointment cost did not affect the results (Table 1).

### Cost estimation of treatment of CIN2+ including follow-up

In the Pap smear arm, 68 women had CIN2+ and in the HPV self-sampling arm, 175 women had CIN2+ (Table 2). The total cost of treatment of histological CIN2+, was € 444,125 (192 treatments) in the HPV self-sampling arm and € 235,211 (70 treatments) in the Pap smear arm. Cost per treated woman was 45% higher in the Pap smear arm (€ 3675) than in the HPV self-sampling arm (€ 2538) (Table 2).

**Table 2:** Resources required for treatment including further preoperative assessment and follow-up of CIN2+ among women by intervention group with associated costs in 2019 (€ 1 = 10.5912 SEK)

		**Pap smear**	**HPV self-sampling**		
	**Unit Cost (€)**	**Units**	**Costs (€)**	**Units**	**Costs (€)**
CIN2+ histological analysis	775	68	52,700	175	135,625
*Pregnant*	*0*	*10*	*0*	*11*	*0*
**Treatment**					
Excision (local anesthesia)	753	40	30,120	151	113,703
Excision (general anesthesia)	1767	23	40,641	33	58,311
Hysterectomy (mini-invasive)	5825	3	17,475	5	29,125
Hysterectomy/trachelectomy (radical)	11,973	4	47,893	3	35,919
**Total number of treatments**		**70**		**192**	
Inpatient care (mean)	968	24	23,232	39	37,752
**Radiology**					
Abdominal CT scan	145	5	725	7	1015
Thorax CT scan	131	5	655	5	655
Pelvic MR scan	339	4	1356	3	1017
Multidisciplinary meeting (primary)	2647	6	15,882	10	26,470
Multidisciplinary meeting (repeated)	1511	3	4533	3	4533
**Treatment cost**					
**Total cost**			**235,211**		**444,125**
**Treated women**		**64**		**175**	
**Cost per treated woman**			**3675**		**2538**
**Follow-up after treatment**					
Midwife appointment	98	34	3332	89	8722
Colposcopy appointment	470	61	28,670	141	66,270
Biopsy histological analysis	147	22	3234	90	13,230
Pap smear cytological analysis	25	87	2175	226	5650
HPV test	38	72	2736	191	7258
**Total cost incl follow-up**			**275,358**		**545,255**
**Treated women**		**64**		**175**	
**Cost per treated woman incl follow-up**			**4302**		**3116**

## Discussion

This study demonstrated that repeated self-sampling for HPV testing at home was more effective in increasing participation and detecting CIN2+ and less costly than midwife-collected Pap smear cytology in primary cervical screening. Our results concerning the cost of cervical screening based on self-sampling for HPV testing are in line with those of previous studies modeling the cost-effectiveness of HPV testing in primary cervical screening. In a study from Canada it was concluded that using HPV testing both in primary screening or as triage of equivocal Pap smear results was more effective and cost-effective relative to cytology [21]. In a study from the Netherlands it was predicted that replacing cytology in primary screening by way of HPV testing and cytology triage would increase the total cost, but this could be compensated for by extended screening intervals [22]. A study from Australia, including a vaccinated population, showed that primary HPV testing with partial genotyping of HPV16/18 every 5 years was a more effective and less costly strategy than cytology screening every 2 years [23]. In triage of cytological ASCUS or LSIL, genotyping for HPV16/18 has shown to be the most cost-effective strategy [24].

Self-sampling for HPV testing is one of the most effective (in improving participation) and cost-effective interventions as regards non-responders, and has been evaluated in several European populations [25–29]. Similar to our study results, previous intervention studies for sensitivity analysis on participation rate, health care costs, Pap smears and self-sampling kits have not affected the results. Our published randomized study was the first to demonstrate an increase in participation in *primary screening* with vaginal self-sampling using PCR-based HPV test, as compared with midwife-collected Pap smear cytology [18]. In the present study we provide a cost-effectiveness analysis of the randomized trial. The total cost per woman participating in primary screening was 4.2 times higher in the Pap smear arm than in the HPV self-sampling arm.

The estimation of treatment costs showed that the cost *per treated woman* was 45% higher in the Pap smear arm, since more women with cervical intraepithelial neoplasia grade 2 (CIN2) were detected by HPV self-sampling than by Pap smears. This, together with somewhat different treatment policies in different hospitals, resulted in more excisions under local anesthesia and proportionally fewer hysterectomies in the HPV self-sampling arm than in the Pap smear arm, resulting in a lower cost per treated woman. During the study period about 50% of all women visited a gynecologist for colposcopy as follow-up after treatment (‘test of cure’). According to present guidelines the recommended ‘test of cure’ is cytology and HPV testing based on a sample collected by a midwife. As previous studies have showed that only up to about 30% of patients are HPV-positive 6 months after treatment [30–32], only these might need a colposcopy, resulting in lower costs for the HPV self-sampling arm.

One strength of our CEA is that we retrospectively collected healthcare events for all included patients in a randomized trial. We then applied the direct medical costs reported from the financial records, together with costs of self-sampling kits and postal fees to each individual patient. The data therefore provide reliable estimates of costs.

This study included all direct medical costs, but not all direct costs (e.g. transportation costs to and from the clinic, parking fees or childcare costs) and indirect costs (i.e. those corresponding to the ‘time off work’ needed for scheduling and conducting the screening appointment) related to clinician-collected samples. These costs can be substantial [33] and including them would result in a more comprehensive estimate of the actual differences in costs between alternative strategies for primary cervical screening. ‘Time off work’ can also represent a barrier to attending clinic-based screening, and avoiding such barriers thus might lead to higher population coverage [33]. In our previous CEA study we compared self-sampling for HPV testing with Pap smear cytology using a Markov model simulating the natural history of cervical cancer, plus empirical data to create a Swedish female cohort [34]. We concluded that self-sampling for HPV testing is cost-effective every 5 years among women aged over 35 years compared with cytology-based screening with Pap smears [34].

Swedish national guidelines on primary cervical screening with HPV testing and triage with liquid-based cytology among women aged over 30 years have recently been published [35, 36]. The future societal costs are estimated to decrease as a result of fewer cancer cases needing healthcare, and the near-time healthcare costs of the screening program are estimated to increase as a result of more cases needing colposcopy, treatment and follow-up (https://www.socialstyrelsen.se/globalassets/sharepoint-dokument/artikelkatalog/nationella-screeningprogram/2019-4-12.pdf). It is therefore of interest to assess alternative screening strategies that could both increase the participation in screening and reduce the overall costs and women suffering. This CEA on repeated self-sampling for HPV testing shows profitable results with respect to increasing participation at lower cost than conventional cytology in primary cervical screening.

## Conclusions

The choice of a test, in addition to cost, is also highly influenced by the tests clinical performance and acceptance by women. Our earlier randomized study showed higher participation rate and the present study, based on the same population, shows reduced costs with home-based repeated self-sampling for HPV testing in comparison with clinic-based Pap smear sampling. Validated PCR-based HPV tests have shown good performance based on self-samples, but further studies are needed to evaluate the performance of different self-sampling kits, sample handling logistics, acceptance and costs, to guide policy-makers on the use of self-sampling for HPV testing in primary cervical screening.

## Data Availability

All data generated or analyzed during this study are included in this published article and in a previously published article [18].

## Abbreviations

AIS: Adenocarcinoma in situ
ASCUS: Atypical squamous cells of unknown significance
CEA: Cost-effectiveness analysis
CIN1: Low-grade cervical intraepithelial neoplasia
CIN2 +: Cervical intraepithelial neoplasia grade 2 or more
HPV: Human papillomavirus
ICER: Incremental cost-effectiveness ratios
LEEP: Loop electrosurgical excision procedure
TZ3: Transformation zone 3
